# A minimally invasive technique for closing an iatrogenic subclavian artery cannulation using the Angio-Seal closure device: two case reports

**DOI:** 10.1186/1752-1947-6-82

**Published:** 2012-03-09

**Authors:** Peter L Szkup

**Affiliations:** 1 Department of Medical Imaging, Royal University Hospital University of Saskatchewan, 103 Hospital Drive, Saskatoon, SK S7N 0W8, Canada

## Abstract

**Introduction:**

In the two cases described here, the subclavian artery was inadvertently cannulated during unsuccessful access to the internal jugular vein. The puncture was successfully closed using a closure device based on a collagen plug (Angio-Seal, St Jude Medical, St Paul, MN, USA). This technique is relatively simple and inexpensive. It can provide clinicians, such as intensive care physicians and anesthesiologists, with a safe and straightforward alternative to major surgery and can be a life-saving procedure.

**Case presentation:**

In the first case, an anesthetist attempted ultrasound-guided access to the right internal jugular vein during the preoperative preparation of a 66-year-old Caucasian man. A 7-French (Fr) triple-lumen catheter was inadvertently placed into his arterial system. In the second case, an emergency physician inadvertently placed a 7-Fr catheter into the subclavian artery of a 77-year-old Caucasian woman whilst attempting access to her right internal jugular vein. Both arterial punctures were successfully closed by means of a percutaneous closure device (Angio-Seal). No complications were observed.

**Conclusions:**

Inadvertent subclavian arterial puncture can be successfully managed with no adverse clinical sequelae by using a percutaneous vascular closure device. This minimally invasive technique may be an option for patients with non-compressible arterial punctures. This report demonstrates two practical points that may help clinicians in decision-making during daily practice. First, it provides a practical solution to a well-known vascular complication. Second, it emphasizes a role for proper vascular ultrasound training for the non-radiologist.

## Introduction

Inadvertent puncture of the subclavian artery during placement of a central venous catheter is a well-known complication occurring in the operating room, intensive care unit (ICU) and emergency setting. Owing to its non-compressible location, subclavian arterial cannulation may result in hemorrhage (which may be fatal) as the catheter is removed. Two cases of successful percutaneous closure of a puncture in a subclavian artery, using a closure device based on a collagen plug (Angio-Seal, St. Jude Medical, St. Paul, MN, USA), are reported. This technique is minimally invasive and relatively inexpensive. It can provide clinicians, such as ICU physicians and anesthesiologists, with a safe and straightforward alternative to major surgery and can be a life-saving procedure.

This report demonstrates two practical points that may help clinicians in decision-making during daily practice. First, it provides a practical solution to a well-known vascular complication. Second, it emphasizes a role for proper vascular ultrasound training for the non-radiologist.

## Case presentation

### Case one

During preoperative preparation for posterior cervical decompression in a 66-year-old Caucasian man, an anesthesiologist used ultrasound to access our patient's right internal jugular vein (IJV) to place a venous catheter. After what was presumed to be successful ultrasound-guided venous access, a 7-Fr triple-lumen catheter was inserted. The anesthesiologist noted an arterial waveform arising from the catheter tracing. The catheter was left *in situ *out of concern that removing it could lead to uncontrollable bleeding, without the ability to directly compress the arterial puncture. The surgical procedure was postponed.

**Figure 1:**
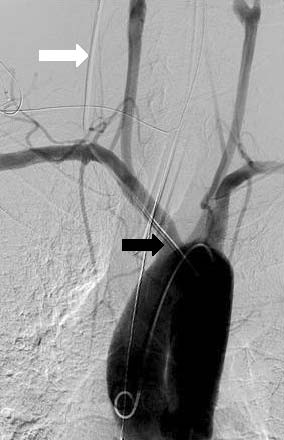
**Angiogram of the aortic arch in Case 1 showing a misplaced central venous catheter (white arrow) entering the arterial system beyond the origin of the right vertebral artery**. The tip of the catheter is in the aortic arch (black arrow).

Our patient was transferred from the operating room to the angiography suite and remained under the general anesthetic. Angiography via the misplaced catheter revealed a flow of contrast in the aortic arch because of the arterial position of the catheter. The decision was made to perform a formal angiogram via his femoral artery to better visualize the catheter entry point into his arterial system. Access through his femoral artery was achieved. An angiogram showed the entry point of the misplaced catheter in his proximal right subclavian artery, just beyond the origin of his right vertebral artery (Figure 1). An angiogram of his right vertebral artery revealed a dominant vessel with prominent spinal arterial feeders. A consultation with the cardiothoracic surgeon revealed that, despite the proximal entry point of the misplaced catheter into the subclavian artery, the surgeon would not be able to directly visualize or palpate the artery during surgical exploration. Placement of a covered stent was an attractive alternative. An analysis of the images acquired determined that the dominant right vertebral artery would probably be occluded by the placement of a covered stent and so it was determined that our patient was not a good candidate for the safe placement of a covered stent.

**Figure 2:**
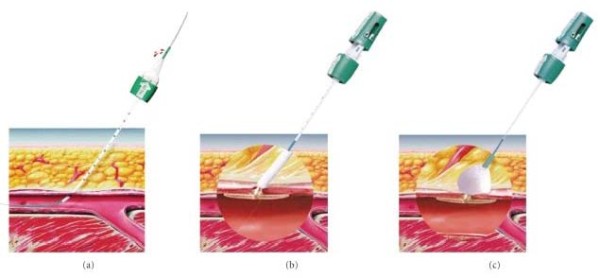
**Closure device**. **(A) **Introduction of a guidewire into the artery. (**B,C) **Insertion of a bioabsorbable anchor and collagen sponge.

**Figure 3:**
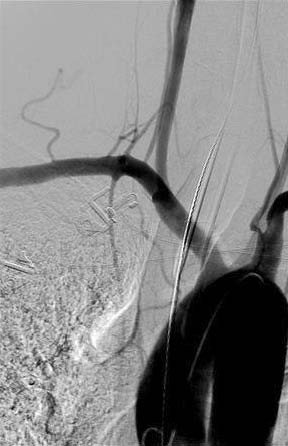
**Angiogram of the aortic arch in Case 1 after insertion of a closure device, showing no contrast extravasation or vessel narrowing**.

The decision was made to remove the catheter and close the puncture site with a percutaneous vascular closure device (Figure 2). A 0.035 Amplatz guidewire (Cook, Bloomington, IN, USA) was inserted through the misplaced catheter, with the tip of the wire in the descending thoracic aorta. The misplaced venous catheter was removed, and the closure device was placed. Satisfactory hemostasis was achieved immediately. A control angiogram confirmed that there was no extravasation of contrast and his subclavian and right vertebral arteries had normal patency (Figure 3). Our patient was transported to the recovery room and, later, to the ward.

### Case two

**Figure 4:**
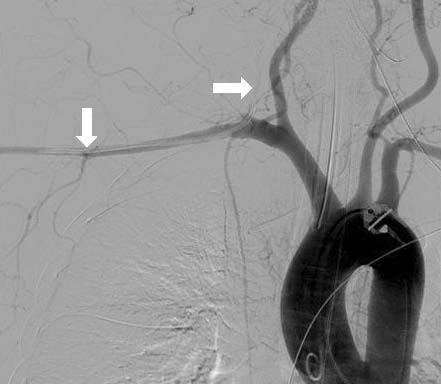
**Angiogram of the aortic arch in Case 2 showing a misplaced central venous catheter in the right subclavian artery (arrows)**.

A 77-year-old Caucasian woman was successfully resuscitated after cardiac arrest due to a pulmonary embolism. The emergency physician attempted to insert a cannula into her right IJV by using an anatomic landmark technique with a 7-Fr catheter. Pulsating blood was observed and an arterial puncture suspected. A catheter was secured to her skin and our patient was transferred from the ICU to the angiography suite. An aortic angiogram was performed using access via her femoral artery. This confirmed the position of a misplaced central venous catheter within her right subclavian artery. The misplaced catheter entered through the proximal subclavian artery, just beyond the origin of the right vertebral artery, as in Case 1. The tip of the catheter was near the origin of an axillary artery (Figure 4). A second catheter was used to snare the misplaced catheter into her aortic arch. A closure device was deployed in the standard fashion. Satisfactory hemostasis was achieved immediately. Normal arterial patency and the absence of extravasation of contrast were confirmed by a control angiogram. Our patient was transferred to our ICU in stable condition.

## Discussion

**Figure 5:**
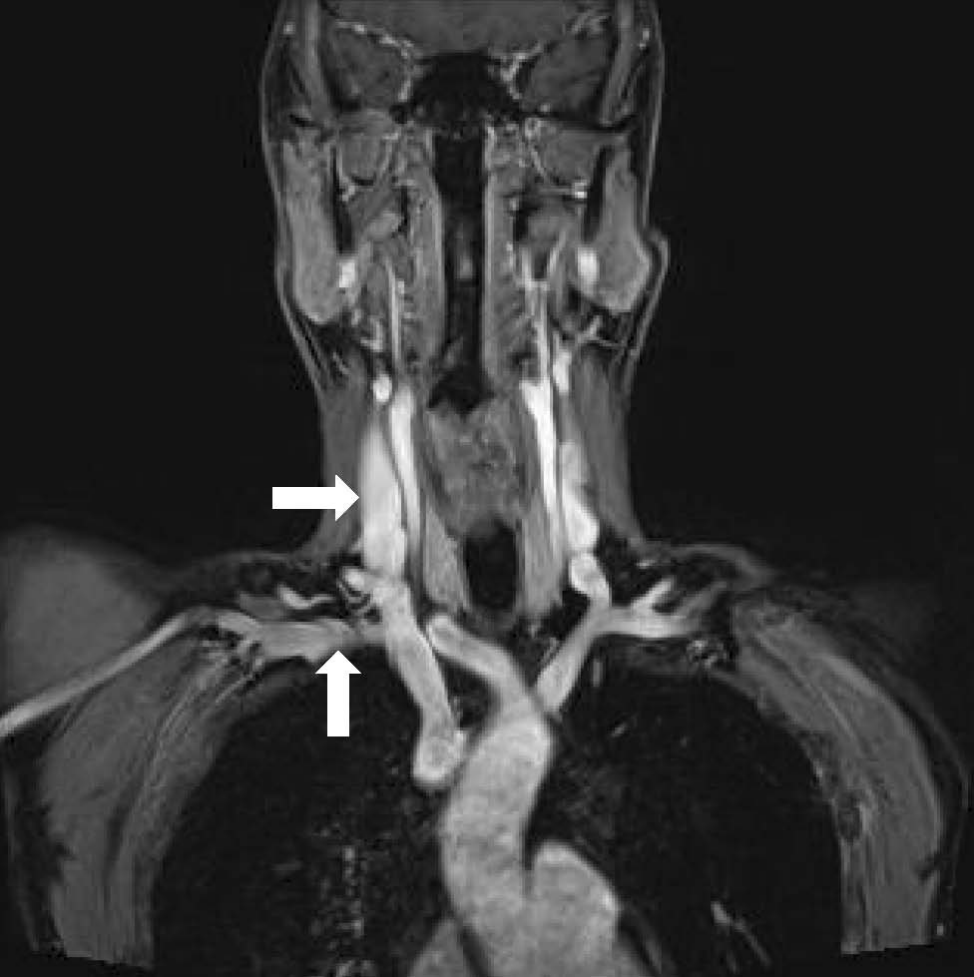
**A magnetic resonance angiogram from a different patient showing the right subclavian artery (vertical arrow) in relation to the right internal jugular vein (horizontal arrow)**.

Inadvertent carotid artery puncture during placement of a central venous catheter is a well-known complication and has a reported incidence of up to 3.7% [1,2]. Complications caused by subclavian artery puncture are relatively rare. Their actual incidence and frequency are unknown because cases are probably under-reported. Almost all subclavian artery injuries reported in the literature are right-sided. It has been supposed that this complication is a right-sided phenomenon because of the specific anatomic layout [3]. The right subclavian artery branches from the innominate artery medial and posterior to the distal right IJV, whereas the left subclavian artery arises from the aortic arch and turns laterally to the IJV (Figure 5). This relationship is further supported by the fact that all reported cases of subclavian artery injury (including the present ones) demonstrated a typical location of the injury corresponding to the proximal part of the subclavian artery.

The arterial injury in Case 1 could have been caused by a needle inserted too far and too low, transversing the IJV and entering an artery, or by a stiff dilator. Despite a properly placed guidewire, a stiff vascular dilator may go over the soft wire and through the vein wall, causing injury to the adjacent artery. This illustrates two important practical points. First, an IJV puncture should be made closer to the apex of the two heads of the sternocleidomastoid muscle. Second, the dilator should be used to dilate skin and subcutaneous tissue rather than the wall of a vein.

The best ways to prevent arterial perforation during central venous catheter insertion are to use real-time ultrasonography and to recognize that the vessel the needle is entering is actually a vein. The use of real-time ultrasound guidance has been promoted as a method for reducing the risk of complications during central venous catheterization [1,4,5]. During IJV catheterization, ultrasound guidance reduces the number of mechanical complications, the number of catheter placement failures and the time required for insertion [6]. In one study, the success rate with real-time ultrasound guidance was 100%, compared with 88% when ultrasound was not used. The same study showed that the incidence of carotid puncture was reduced from 8.3% to 1.7% and that the average access time was reduced by a factor of four [7]. The goals of ultrasound guidance are to enter the skin at an appropriate location and to directly visualize the needle tip entering the vessel. This is a very operator-dependent process, and success depends upon the ability of the operator to appropriately align the ultrasound probe and the exploring needle so that the needle can be seen continuously during the procedure. As with all new techniques, ultrasound-guided catheterization requires training. Chapman *et al. *[8] have written an excellent review article pertaining to the practical aspects of needle visualization during ultrasound-guided procedures.

Iatrogenic trauma to the carotid or subclavian arteries may cause fatal bleeding, arterial dissection, emboli, thrombosis, pseudoaneurysm, arteriovenous fistula and airway obstruction [1,9,10]. In the anesthetized patient, inadvertent arterial cannulation that is not promptly recognized and managed can lead to debilitating irreversible complications. Although misplaced venous catheters may be removed with direct compression, some arterial sites, such as the subclavian artery, are non-compressible because of their anatomic location. Removing the catheter with a 'wait-and-see' strategy is no longer recommended these days, given the previously mentioned documented complications and the availability of minimally invasive techniques.

Several options have been reported in the literature in the management of inadvertent subclavian arterial cannulation. Surgical repair usually requires partial removal of the first rib or a thoracotomy. Endovascular placement of a covered stent provides an elegant and efficient form of treatment [1,11]. Unfortunately, in both our cases, there was a risk of occluding the dominant vertebral artery after stent deployment. Local percutaneous treatment has recently been reported for inadvertent arterial puncture [2,12,13].

The closure device used in our cases creates a mechanical seal by sandwiching the arteriotomy between a bioabsorbable anchor and a collagen sponge, which dissolve within 60 to 90 days. It is important to have angiographic confirmation of a safe landing zone before deploying the device and to prevent side-branch compromise. Prior to insertion of the closure device, it is important to make sure that the introducing point of the sheath is away from the origins of the vertebral and other large artery branches. Use of the closure device is contraindicated at bifurcations and in small vessels. This device is most commonly used to restore hemostasis after angiography in a femoral artery. It is recommended for use after insertion of catheters up to 8-Fr but has been used successfully with 10-Fr arteriotomies [2]. Treatment of a large-artery injury, including successful treatment of a descending thoracic aorta after inadvertent placement of a 12-Fr drainage catheter, has also been reported [14].

Possible adverse events for vascular closure devices include, but are not limited to, bleeding, arteriovenous fistula, pseudoaneurysm, allergic reaction and infection. Infection associated with vascular closure device placement is uncommon but is an extremely serious complication. Morbidity is high, and aggressive medical and surgical interventions are required to achieve a positive outcome. The high morbidity associated with infection indicates the need to identify patients who are at increased risk of these complications, such as those with immunodepression, diabetes, or obesity [15]. The most feared complication of vascular closure devices is acute limb ischemia, requiring emergent surgical or endovascular intervention. This can occur secondary to embolization, thrombosis or occlusion from the intravascular component of the device [16].

## Conclusions

Inadvertent subclavian arterial puncture can be successfully managed with no adverse clinical sequelae by using a percutaneous vascular closure device. The technique is relatively straightforward, especially for those who are familiar with the device. It is no longer necessary to simply remove the catheter in a 'wait and see' strategy as the arteriotomy can be rapidly and successfully treated using a relatively inexpensive arterial closure device.

## Abbreviations

Fr: French; ICU: intensive care unit; IJV: internal jugular vein.

## Consent

Written informed consents were obtained from the patients for publication of these case reports and any accompanying images. A copy of the written consent is available for review by the Editor-in-Chief of this journal.

## Competing interests

The authors declare that they have no competing interests.
